# 4-Methyl-*N*-(2-phenyl­eth­yl)-2-propyl-1*H*-benzimidazole-6-carboxamide

**DOI:** 10.1107/S1600536812036707

**Published:** 2012-09-01

**Authors:** Jin-Liang Wang, Wei-Fa Yu

**Affiliations:** a R&D Center for Pharmaceuticals, School of Chemical Engineering & the Environment, Beijing Institute of Technology, Beijing 100083, People’s Republic of China; b SINOPEC Research Institute of Petroleum Processing, Beijing 100083, People’s Republic of China

## Abstract

There are two independent mol­ecules in the asymmetric unit of the title compound, C_20_H_23_N_3_O, in which the dihedral angles between the phenyl ring of the phenyl­ethyl­amino group and the benzimidazole system are 73.98 (15) and 15.93 (16)°. The crystal packing features N—H⋯O and N—H⋯N hydrogen bonds.

## Related literature

For the background to the title compound and its derivatives, see Mahiuddin *et al.* (2007 ▶); Namrata *et al.* (2012 ▶); Zhang *et al.* (2012 ▶); For hydrogen bonding, see: Desiraju (1995 ▶).
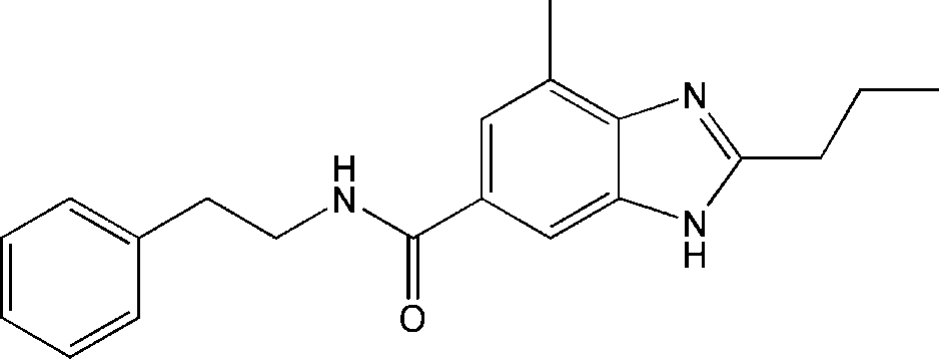


## Experimental

### 

#### Crystal data

C_20_H_23_N_3_O
*M* *_r_* = 321.41
Triclinic, 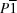

*a* = 10.2028 (7) Å
*b* = 11.9126 (9) Å
*c* = 17.0553 (17) Å
α = 101.563 (4)°
β = 99.110 (4)°
γ = 113.808 (2)°
*V* = 1790.6 (3) Å^3^
*Z* = 4
Mo *K*α radiation
μ = 0.08 mm^−1^
*T* = 296 K
0.41 × 0.30 × 0.20 mm

#### Data collection

Bruker APEXII CCD area-detector diffractometer
Absorption correction: multi-scan (*SADABS*; Bruker, 2007 ▶) *T*_min_ = 0.670, *T*_max_ = 0.746
17517 measured reflections
6081 independent reflections
4250 reflections with *I* > 2σ(*I*)
*R*_int_ = 0.029

#### Refinement

*R*[*F* ^2^ > 2σ(*F* ^2^)] = 0.049
*wR*(*F* ^2^) = 0.155
*S* = 1.08
6081 reflections
450 parameters
46 restraints
H atoms treated by a mixture of independent and constrained refinement
Δρ_max_ = 0.37 e Å^−3^
Δρ_min_ = −0.30 e Å^−3^

### 

Data collection: *APEX2* (Bruker 2007 ▶); cell refinement: *APEX2* and *SAINT* (Bruker 2007 ▶); data reduction: *SAINT*; program(s) used to solve structure: *SHELXS97* (Sheldrick, 2008 ▶); program(s) used to refine structure: *SHELXL97* (Sheldrick, 2008 ▶); molecular graphics: *SHELXTL* (Sheldrick, 2008 ▶); software used to prepare material for publication: *SHELXTL* and *PLATON* (Spek, 2009 ▶).

## Supplementary Material

Crystal structure: contains datablock(s) I, global. DOI: 10.1107/S1600536812036707/zj2085sup1.cif

Structure factors: contains datablock(s) I. DOI: 10.1107/S1600536812036707/zj2085Isup2.hkl

Supplementary material file. DOI: 10.1107/S1600536812036707/zj2085Isup3.cml

Additional supplementary materials:  crystallographic information; 3D view; checkCIF report

## Figures and Tables

**Table 1:** Hydrogen-bond geometry (Å, °)

*D*—H⋯*A*	*D*—H	H⋯*A*	*D*⋯*A*	*D*—H⋯*A*
N1—H1⋯O2^i^	0.88	1.91	2.744 (2)	157
N4—H4⋯N2^ii^	0.89	2.01	2.882 (3)	165
N5—H5⋯O1	0.88	2.06	2.849 (4)	148
N6—H6⋯N3^iii^	0.88	2.25	3.109 (2)	169

Symmetry codes: (i) 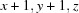; (ii) 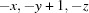; (iii) 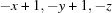.

**Fig. 1.**
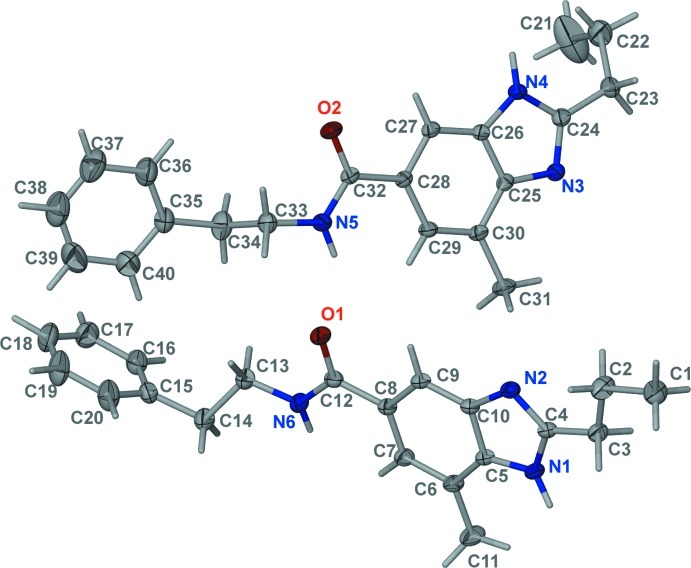
The molecular structure of the title compound with displacement ellipsoids drawn at the 50% probability level. H atoms are presented as small spheres of the arbitrary radius.

**Fig. 2.**
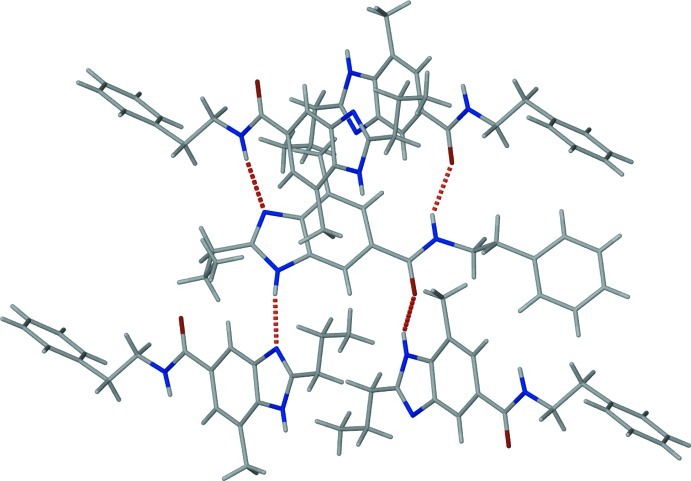
The special show of the intermolecular H-bonding in the crystal structure.

**Fig. 3.**
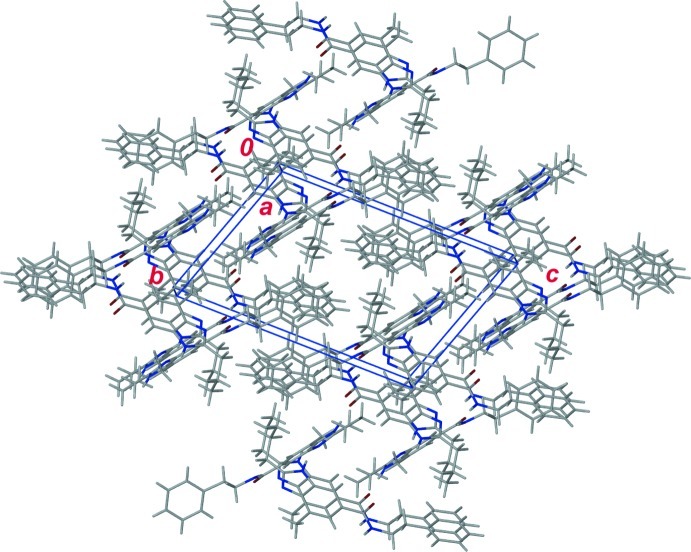
The packing structure of the title compound, all hydrogen-bonding interactions are omitted for clarity.

## supplementary crystallographic information

### Comment

Benzimidazole is an important scaffold with biological activities generally ultilized in antihypertensive drugs, proton pump inhibitors and antimicrobial agents, etc. (Mahiuddin *et al.*, 2007.; Namrata *et al.*, 2012). As a part of our study of 6-substituted carbamoyl benzimidazoles as a nonpeptidic angiotensin II AT1 receptor antagonist (Zhang *et al.*, 2012), herein we report the synthesis and crystal structure of the title compound of this family. In the asymmetric unit of the title compound, there are two independent molecules with different dihedral angles between the phenyl ring of the phenylethylamino and the benzimidazole ring. In the O1(carbonyl)-containing molecule, the dihedral angle between the phenyl ring of the phenylethylamino and the benzimidazole ring is -1.8 (5) °, while the O2(carbonyl)-containing one has the value of -1.0 (5) ° (Fig. 1). Intermolecular N—H···O and N—H···N hydrogen-bonding (Desiraju, 1995) interactions (Table 1 and Fig. 2,3) are found to stabilize the whole packing structure of the title compound. As shown in Fig.2, each one O2-containing molecule is surrounded by four O1-containing ones through H-bonding interactions.

### Experimental

A suspension of 4-methyl-2-propyl-1H-benzimidazole-6-carboxylic acid (2.18 g, 10 mmol) in thionyl chloride (20 ml, 276 mmol) was refluxed for 2 h, and then the excess thionyl chloride was removed under reduced pressure to provide the crude acid chloride as an off-white solid. The crude product was used in the next step without further purification. To a stirred suspension of above acid chloride in 100 ml of dichloromethane at 293 K was added triethylamine (1.52 g, 15 mmol) dropwise, followed by a solution of phenylethylamine (10 mmol) in 10 ml of dichloromethane. The resulted mixture was stirred overnight and then was filtered, the filtrate was washed with brine and dried over anhydrous sodium sulphate, then was filtered and concentrated. The residue was purified by column chromatography (ethyl acetate–methanol, 4:1 v/v) to afford the title compound as a white solid, yield 72%, m.p. 425 ~ 427 K; Single crystals suitable for X-ray diffraction were prepared by slow evaporation of a solution of the title compound in ethanol at room temperature.

### Refinement

The completeness of the final refinement is less than 97 percent due to the deficient diffraction on high degree.All H atoms were discernible in the difference electron density maps.Nevertheless, the hydrogen atoms were placed into idealized positions and allowed to ride on their respective carrier atoms, with C—H = 0.93 for aryl, 0.96 Å and 0.97 Å for methyl and methylene H atoms, respectively. The N—H bonds were defined with constraint refinement at 0.88 Å. *U*_iso_(H) = 1.2*U*_eq_(C)_aryl_/_methylene_/_methyl_.

### Crystal data

**Table:**

C_20_H_23_N_3_O	*Z* = 4
*M**_r_* = 321.41	*F*(000) = 688
Triclinic, *P*1	*D*_x_ = 1.192 Mg m^−^^3^
Hall symbol: -P 1	Mo *K*α radiation, λ = 0.71073 Å
*a* = 10.2028 (7) Å	Cell parameters from 362 reflections
*b* = 11.9126 (9) Å	θ = 2.0–25.0°
*c* = 17.0553 (17) Å	µ = 0.08 mm^−^^1^
α = 101.563 (4)°	*T* = 296 K
β = 99.110 (4)°	Block, colorless
γ = 113.808 (2)°	0.41 × 0.30 × 0.20 mm
*V* = 1790.6 (3) Å^3^	

### Data collection

**Table:**

Bruker APEXII CCD area-detector diffractometer	6081 independent reflections
Radiation source: fine-focus sealed tube	4250 reflections with *I* > 2σ(*I*)
Graphite monochromator	*R*_int_ = 0.029
ω scans	θ_max_ = 25.0°, θ_min_ = 2.0°
Absorption correction: multi-scan (*SADABS*; Bruker, 2007)	*h* = −11→12
*T*_min_ = 0.670, *T*_max_ = 0.746	*k* = −14→13
17517 measured reflections	*l* = −17→20

### Refinement

**Table:**

Refinement on *F*^2^	Secondary atom site location: difference Fourier map
Least-squares matrix: full	Hydrogen site location: inferred from neighbouring sites
*R*[*F*^2^ > 2σ(*F*^2^)] = 0.049	H atoms treated by a mixture of independent and constrained refinement
*wR*(*F*^2^) = 0.155	*w* = 1/[σ^2^(*F*_o_^2^) + (0.0753*P*)^2^ + 0.4342*P*] *P* = (*F*_o_^2^ + 2*F*_c_^2^)/3
*S* = 1.08	(Δ/σ)_max_ < 0.001
6081 reflections	Δρ_max_ = 0.37 e Å^−^^3^
450 parameters	Δρ_min_ = −0.30 e Å^−^^3^
46 restraints	Extinction correction: *SHELXL97* (Sheldrick, 2008), Fc^*^=kFc[1+0.001xFc^2^λ^3^/sin(2θ)]^-1/4^
Primary atom site location: structure-invariant direct methods	Extinction coefficient: 0.0088 (17)

### Special details

**Table:**

Geometry. All esds (except the esd in the dihedral angle between two l.s. planes) are estimated using the full covariance matrix. The cell esds are taken into account individually in the estimation of esds in distances, angles and torsion angles; correlations between esds in cell parameters are only used when they are defined by crystal symmetry. An approximate (isotropic) treatment of cell esds is used for estimating esds involving l.s. planes.
Refinement. Refinement of F^2^ against ALL reflections. The weighted R-factor wR and goodness of fit S are based on F^2^, conventional R-factors R are based on F, with F set to zero for negative F^2^. The threshold expression of F^2^ > 2sigma(F^2^) is used only for calculating R-factors(gt) etc. and is not relevant to the choice of reflections for refinement. R-factors based on F^2^ are statistically about twice as large as those based on F, and R- factors based on ALL data will be even larger.

### Fractional atomic coordinates and isotropic or equivalent isotropic displacement parameters (Å^2^)

**Table:**

	*x*	*y*	*z*	*U*_iso_*/*U*_eq_	
H1	0.7674 (19)	1.1106 (15)	0.1249 (14)	0.060 (7)*	
H4	−0.2645 (15)	0.1827 (16)	−0.1485 (14)	0.055 (7)*	
H5	0.2616 (18)	0.460 (2)	0.2126 (15)	0.069 (8)*	
H6	0.7939 (19)	0.598 (2)	0.2321 (15)	0.066 (8)*	
C1	0.3195 (3)	1.2092 (3)	0.08894 (19)	0.0728 (8)	
H1A	0.2239	1.1948	0.0974	0.109*	
H1B	0.3159	1.2026	0.0315	0.109*	
H1C	0.3927	1.2932	0.1227	0.109*	
C2	0.3606 (3)	1.1097 (3)	0.1127 (2)	0.0710 (8)	
H2A	0.3619	1.1157	0.1704	0.085*	
H2B	0.2848	1.0250	0.0793	0.085*	
C3	0.5086 (3)	1.1247 (2)	0.10119 (15)	0.0503 (6)	
H3A	0.5067	1.1194	0.0435	0.060*	
H3B	0.5836	1.2098	0.1345	0.060*	
C4	0.5540 (2)	1.02936 (19)	0.12349 (13)	0.0427 (5)	
C5	0.7011 (2)	0.93810 (19)	0.14869 (13)	0.0407 (5)	
C6	0.8141 (2)	0.9008 (2)	0.15921 (14)	0.0473 (5)	
C7	0.7821 (2)	0.7941 (2)	0.18722 (14)	0.0481 (5)	
H7A	0.8550	0.7666	0.1963	0.058*	
C8	0.6452 (2)	0.72490 (19)	0.20267 (13)	0.0416 (5)	
C9	0.5340 (2)	0.76211 (19)	0.18845 (13)	0.0426 (5)	
H9A	0.4420	0.7163	0.1972	0.051*	
C10	0.5623 (2)	0.86912 (19)	0.16083 (13)	0.0403 (5)	
C11	0.9585 (3)	0.9701 (3)	0.1396 (2)	0.0711 (8)	
H11A	1.0215	0.9301	0.1511	0.107*	
H11B	1.0068	1.0580	0.1731	0.107*	
H11C	0.9398	0.9664	0.0819	0.107*	
C12	0.6116 (2)	0.6129 (2)	0.23587 (13)	0.0437 (5)	
C13	0.6911 (3)	0.4712 (2)	0.29015 (16)	0.0601 (6)	
H13A	0.6338	0.3898	0.2472	0.072*	
H13B	0.6322	0.4768	0.3288	0.072*	
C14	0.8298 (3)	0.4735 (3)	0.33495 (18)	0.0699 (7)	
H14A	0.8900	0.5560	0.3765	0.084*	
H14B	0.8866	0.4625	0.2961	0.084*	
C15	0.7950 (3)	0.3686 (3)	0.37630 (15)	0.0579 (6)	
C16	0.7603 (3)	0.2458 (3)	0.33270 (16)	0.0689 (7)	
H16A	0.7644	0.2289	0.2777	0.083*	
C17	0.7198 (4)	0.1472 (3)	0.3681 (2)	0.0823 (9)	
H17A	0.6971	0.0649	0.3371	0.099*	
C18	0.7128 (4)	0.1698 (4)	0.4486 (2)	0.0923 (10)	
H18A	0.6830	0.1031	0.4725	0.111*	
C19	0.7503 (5)	0.2916 (4)	0.4931 (2)	0.1045 (12)	
H19A	0.7494	0.3084	0.5486	0.125*	
C20	0.7896 (4)	0.3905 (3)	0.45784 (18)	0.0886 (10)	
H20A	0.8127	0.4727	0.4892	0.106*	
C21	−0.2363 (7)	0.0418 (5)	−0.3386 (3)	0.187 (3)	
H21A	−0.3117	−0.0448	−0.3650	0.281*	
H21B	−0.1802	0.0466	−0.2861	0.281*	
H21C	−0.1711	0.0675	−0.3734	0.281*	
C22	−0.3053 (4)	0.1261 (3)	−0.32530 (19)	0.0923 (10)	
H22A	−0.3727	0.0981	−0.2912	0.111*	
H22B	−0.3637	0.1189	−0.3784	0.111*	
C23	−0.1962 (3)	0.2635 (2)	−0.28424 (14)	0.0564 (6)	
H23A	−0.1253	0.2884	−0.3168	0.068*	
H23B	−0.2494	0.3149	−0.2863	0.068*	
C24	−0.1116 (2)	0.2973 (2)	−0.19662 (13)	0.0444 (5)	
C25	0.0603 (2)	0.38852 (19)	−0.08105 (12)	0.0388 (5)	
C26	−0.0660 (2)	0.30365 (19)	−0.06467 (12)	0.0381 (5)	
C27	−0.0696 (2)	0.28931 (19)	0.01351 (13)	0.0412 (5)	
H27A	−0.1556	0.2326	0.0231	0.049*	
C28	0.0598 (2)	0.36249 (18)	0.07695 (12)	0.0382 (5)	
C29	0.1881 (2)	0.44721 (19)	0.06003 (13)	0.0421 (5)	
H29A	0.2743	0.4948	0.1034	0.050*	
C30	0.1921 (2)	0.46316 (19)	−0.01772 (13)	0.0427 (5)	
C31	0.3295 (3)	0.5560 (2)	−0.03364 (15)	0.0644 (7)	
H31A	0.4081	0.5978	0.0169	0.097*	
H31B	0.3093	0.6190	−0.0532	0.097*	
H31C	0.3589	0.5105	−0.0748	0.097*	
C32	0.0549 (2)	0.34704 (19)	0.16112 (13)	0.0420 (5)	
C33	0.1868 (3)	0.3983 (2)	0.30617 (14)	0.0545 (6)	
H33A	0.0883	0.3677	0.3150	0.065*	
H33B	0.2496	0.4812	0.3469	0.065*	
C34	0.2479 (4)	0.3063 (3)	0.31926 (17)	0.0765 (8)	
H34A	0.3500	0.3417	0.3158	0.092*	
H34B	0.1915	0.2267	0.2746	0.092*	
C35	0.2442 (3)	0.2766 (3)	0.40085 (16)	0.0652 (7)	
C36	0.1137 (4)	0.2081 (4)	0.4166 (2)	0.0960 (10)	
H36A	0.0252	0.1813	0.3768	0.115*	
C37	0.1083 (6)	0.1767 (4)	0.4904 (3)	0.1156 (13)	
H37A	0.0175	0.1301	0.5001	0.139*	
C38	0.2369 (7)	0.2147 (4)	0.5481 (2)	0.1115 (13)	
H38A	0.2346	0.1926	0.5974	0.134*	
C39	0.3665 (6)	0.2835 (4)	0.5348 (2)	0.1176 (14)	
H39A	0.4544	0.3102	0.5751	0.141*	
C40	0.3712 (4)	0.3158 (3)	0.4614 (2)	0.0939 (10)	
H40A	0.4626	0.3650	0.4532	0.113*	
N1	0.6923 (2)	1.03909 (17)	0.12434 (11)	0.0445 (4)	
N2	0.47131 (19)	0.92760 (16)	0.14350 (11)	0.0444 (4)	
N3	0.02963 (19)	0.38310 (16)	−0.16468 (10)	0.0438 (4)	
N4	−0.1741 (2)	0.24659 (17)	−0.13948 (11)	0.0439 (4)	
N5	0.1783 (2)	0.41362 (19)	0.22348 (11)	0.0506 (5)	
N6	0.7166 (2)	0.57415 (18)	0.25232 (12)	0.0502 (5)	
O1	0.49045 (17)	0.55903 (16)	0.25059 (10)	0.0586 (4)	
O2	−0.06237 (17)	0.27366 (15)	0.17288 (9)	0.0585 (5)	

### Atomic displacement parameters (Å^2^)

**Table:**

	*U*^11^	*U*^22^	*U*^33^	*U*^12^	*U*^13^	*U*^23^
C1	0.0683 (18)	0.0739 (18)	0.096 (2)	0.0448 (15)	0.0272 (16)	0.0343 (16)
C2	0.0631 (18)	0.0694 (17)	0.103 (2)	0.0387 (15)	0.0372 (16)	0.0397 (16)
C3	0.0488 (14)	0.0447 (12)	0.0580 (14)	0.0199 (11)	0.0156 (11)	0.0174 (11)
C4	0.0361 (12)	0.0379 (11)	0.0478 (13)	0.0112 (9)	0.0117 (10)	0.0109 (9)
C5	0.0345 (12)	0.0382 (11)	0.0448 (12)	0.0116 (9)	0.0126 (9)	0.0114 (9)
C6	0.0345 (12)	0.0491 (13)	0.0573 (14)	0.0158 (10)	0.0168 (10)	0.0163 (11)
C7	0.0363 (12)	0.0501 (13)	0.0616 (14)	0.0206 (10)	0.0155 (11)	0.0195 (11)
C8	0.0350 (12)	0.0423 (11)	0.0434 (12)	0.0142 (10)	0.0108 (9)	0.0108 (9)
C9	0.0323 (11)	0.0403 (11)	0.0486 (13)	0.0102 (9)	0.0121 (9)	0.0115 (10)
C10	0.0314 (11)	0.0398 (11)	0.0444 (12)	0.0126 (9)	0.0100 (9)	0.0091 (9)
C11	0.0452 (15)	0.0715 (17)	0.112 (2)	0.0267 (13)	0.0388 (15)	0.0443 (16)
C12	0.0375 (13)	0.0449 (12)	0.0429 (12)	0.0142 (10)	0.0098 (10)	0.0109 (10)
C13	0.0540 (16)	0.0580 (15)	0.0710 (17)	0.0239 (12)	0.0123 (13)	0.0305 (13)
C14	0.0584 (17)	0.0790 (18)	0.0801 (19)	0.0301 (15)	0.0177 (14)	0.0418 (15)
C15	0.0525 (15)	0.0703 (17)	0.0542 (15)	0.0282 (13)	0.0100 (12)	0.0270 (13)
C16	0.0774 (19)	0.0803 (19)	0.0532 (16)	0.0353 (16)	0.0220 (14)	0.0254 (14)
C17	0.094 (2)	0.0694 (18)	0.081 (2)	0.0336 (17)	0.0212 (18)	0.0268 (16)
C18	0.106 (3)	0.100 (3)	0.093 (3)	0.048 (2)	0.037 (2)	0.061 (2)
C19	0.160 (4)	0.121 (3)	0.061 (2)	0.075 (3)	0.044 (2)	0.047 (2)
C20	0.132 (3)	0.088 (2)	0.0579 (18)	0.060 (2)	0.0217 (18)	0.0249 (16)
C21	0.290 (8)	0.157 (4)	0.116 (4)	0.163 (5)	−0.035 (4)	−0.024 (3)
C22	0.107 (3)	0.084 (2)	0.0583 (18)	0.024 (2)	0.0059 (17)	0.0165 (16)
C23	0.0521 (15)	0.0667 (16)	0.0455 (14)	0.0215 (13)	0.0105 (11)	0.0195 (12)
C24	0.0408 (13)	0.0484 (12)	0.0463 (13)	0.0195 (11)	0.0152 (10)	0.0168 (10)
C25	0.0373 (12)	0.0401 (11)	0.0453 (12)	0.0186 (10)	0.0178 (10)	0.0171 (9)
C26	0.0308 (11)	0.0402 (11)	0.0423 (12)	0.0142 (9)	0.0103 (9)	0.0132 (9)
C27	0.0331 (12)	0.0430 (11)	0.0480 (13)	0.0137 (10)	0.0158 (10)	0.0179 (10)
C28	0.0372 (12)	0.0366 (10)	0.0426 (12)	0.0154 (9)	0.0144 (10)	0.0150 (9)
C29	0.0344 (12)	0.0400 (11)	0.0446 (12)	0.0102 (9)	0.0108 (9)	0.0116 (9)
C30	0.0348 (12)	0.0406 (11)	0.0476 (13)	0.0101 (10)	0.0153 (10)	0.0135 (10)
C31	0.0487 (15)	0.0639 (15)	0.0549 (15)	−0.0010 (12)	0.0177 (12)	0.0186 (12)
C32	0.0396 (13)	0.0395 (11)	0.0446 (12)	0.0140 (10)	0.0143 (10)	0.0131 (10)
C33	0.0585 (15)	0.0595 (14)	0.0436 (13)	0.0230 (12)	0.0143 (11)	0.0179 (11)
C34	0.098 (2)	0.090 (2)	0.0664 (17)	0.0549 (18)	0.0341 (16)	0.0359 (15)
C35	0.082 (2)	0.0677 (16)	0.0573 (16)	0.0396 (15)	0.0214 (15)	0.0258 (13)
C36	0.091 (2)	0.123 (3)	0.095 (2)	0.051 (2)	0.0331 (19)	0.064 (2)
C37	0.133 (3)	0.136 (3)	0.118 (3)	0.069 (3)	0.068 (3)	0.074 (3)
C38	0.171 (4)	0.109 (3)	0.068 (2)	0.069 (3)	0.039 (3)	0.036 (2)
C39	0.132 (4)	0.116 (3)	0.068 (2)	0.040 (3)	−0.014 (2)	0.017 (2)
C40	0.090 (2)	0.091 (2)	0.077 (2)	0.0233 (19)	0.0057 (18)	0.0263 (18)
N1	0.0353 (11)	0.0393 (10)	0.0570 (11)	0.0118 (8)	0.0165 (9)	0.0175 (9)
N2	0.0345 (10)	0.0428 (10)	0.0552 (11)	0.0156 (8)	0.0128 (8)	0.0159 (8)
N3	0.0399 (11)	0.0485 (10)	0.0434 (10)	0.0167 (9)	0.0154 (8)	0.0178 (8)
N4	0.0312 (10)	0.0492 (11)	0.0459 (11)	0.0112 (9)	0.0117 (9)	0.0168 (9)
N5	0.0405 (12)	0.0593 (12)	0.0432 (11)	0.0121 (10)	0.0124 (9)	0.0186 (9)
N6	0.0462 (12)	0.0554 (11)	0.0574 (12)	0.0239 (10)	0.0200 (10)	0.0267 (9)
O1	0.0395 (9)	0.0636 (10)	0.0750 (11)	0.0172 (8)	0.0192 (8)	0.0349 (9)
O2	0.0475 (10)	0.0584 (10)	0.0510 (10)	0.0025 (8)	0.0183 (8)	0.0197 (8)

### Geometric parameters (Å, º)

**Table:**

C1—C2	1.511 (4)	C21—H21B	0.9600
C1—H1A	0.9600	C21—H21C	0.9600
C1—H1B	0.9600	C22—C23	1.494 (4)
C1—H1C	0.9600	C22—H22A	0.9700
C2—C3	1.498 (3)	C22—H22B	0.9700
C2—H2A	0.9700	C23—C24	1.487 (3)
C2—H2B	0.9700	C23—H23A	0.9700
C3—C4	1.481 (3)	C23—H23B	0.9700
C3—H3A	0.9700	C24—N3	1.326 (3)
C3—H3B	0.9700	C24—N4	1.357 (3)
C4—N2	1.315 (3)	C25—C26	1.392 (3)
C4—N1	1.366 (3)	C25—N3	1.394 (3)
C5—N1	1.380 (3)	C25—C30	1.399 (3)
C5—C6	1.393 (3)	C26—N4	1.381 (3)
C5—C10	1.395 (3)	C26—C27	1.382 (3)
C6—C7	1.382 (3)	C27—C28	1.383 (3)
C6—C11	1.500 (3)	C27—H27A	0.9300
C7—C8	1.406 (3)	C28—C29	1.408 (3)
C7—H7A	0.9300	C28—C32	1.490 (3)
C8—C9	1.380 (3)	C29—C30	1.381 (3)
C8—C12	1.488 (3)	C29—H29A	0.9300
C9—C10	1.385 (3)	C30—C31	1.500 (3)
C9—H9A	0.9300	C31—H31A	0.9600
C10—N2	1.397 (3)	C31—H31B	0.9600
C11—H11A	0.9600	C31—H31C	0.9600
C11—H11B	0.9600	C32—O2	1.240 (2)
C11—H11C	0.9600	C32—N5	1.333 (3)
C12—O1	1.235 (2)	C33—N5	1.451 (3)
C12—N6	1.340 (3)	C33—C34	1.499 (4)
C13—N6	1.449 (3)	C33—H33A	0.9700
C13—C14	1.486 (3)	C33—H33B	0.9700
C13—H13A	0.9700	C34—C35	1.505 (4)
C13—H13B	0.9700	C34—H34A	0.9700
C14—C15	1.503 (3)	C34—H34B	0.9700
C14—H14A	0.9700	C35—C36	1.355 (4)
C14—H14B	0.9700	C35—C40	1.363 (4)
C15—C16	1.371 (4)	C36—C37	1.386 (5)
C15—C20	1.377 (4)	C36—H36A	0.9300
C16—C17	1.373 (4)	C37—C38	1.352 (6)
C16—H16A	0.9300	C37—H37A	0.9300
C17—C18	1.364 (4)	C38—C39	1.329 (6)
C17—H17A	0.9300	C38—H38A	0.9300
C18—C19	1.359 (5)	C39—C40	1.385 (5)
C18—H18A	0.9300	C39—H39A	0.9300
C19—C20	1.375 (4)	C40—H40A	0.9300
C19—H19A	0.9300	N1—H1	0.883 (10)
C20—H20A	0.9300	N4—H4	0.892 (10)
C21—C22	1.444 (6)	N5—H5	0.879 (10)
C21—H21A	0.9600	N6—H6	0.875 (10)
			
C2—C1—H1A	109.5	C21—C22—H22A	108.9
C2—C1—H1B	109.5	C23—C22—H22A	108.9
H1A—C1—H1B	109.5	C21—C22—H22B	108.9
C2—C1—H1C	109.5	C23—C22—H22B	108.9
H1A—C1—H1C	109.5	H22A—C22—H22B	107.7
H1B—C1—H1C	109.5	C24—C23—C22	117.0 (2)
C3—C2—C1	113.1 (2)	C24—C23—H23A	108.0
C3—C2—H2A	109.0	C22—C23—H23A	108.0
C1—C2—H2A	109.0	C24—C23—H23B	108.0
C3—C2—H2B	109.0	C22—C23—H23B	108.0
C1—C2—H2B	109.0	H23A—C23—H23B	107.3
H2A—C2—H2B	107.8	N3—C24—N4	112.61 (19)
C4—C3—C2	115.24 (19)	N3—C24—C23	124.40 (19)
C4—C3—H3A	108.5	N4—C24—C23	122.9 (2)
C2—C3—H3A	108.5	C26—C25—N3	109.70 (18)
C4—C3—H3B	108.5	C26—C25—C30	120.55 (18)
C2—C3—H3B	108.5	N3—C25—C30	129.74 (18)
H3A—C3—H3B	107.5	N4—C26—C27	131.64 (18)
N2—C4—N1	112.09 (19)	N4—C26—C25	105.57 (17)
N2—C4—C3	126.25 (19)	C27—C26—C25	122.78 (19)
N1—C4—C3	121.65 (19)	C26—C27—C28	117.46 (18)
N1—C5—C6	132.18 (19)	C26—C27—H27A	121.3
N1—C5—C10	105.01 (18)	C28—C27—H27A	121.3
C6—C5—C10	122.79 (19)	C27—C28—C29	119.67 (19)
C7—C6—C5	115.18 (19)	C27—C28—C32	117.12 (18)
C7—C6—C11	122.6 (2)	C29—C28—C32	123.21 (19)
C5—C6—C11	122.2 (2)	C30—C29—C28	123.3 (2)
C6—C7—C8	123.4 (2)	C30—C29—H29A	118.4
C6—C7—H7A	118.3	C28—C29—H29A	118.4
C8—C7—H7A	118.3	C29—C30—C25	116.26 (18)
C9—C8—C7	119.63 (19)	C29—C30—C31	122.2 (2)
C9—C8—C12	116.51 (19)	C25—C30—C31	121.58 (19)
C7—C8—C12	123.85 (19)	C30—C31—H31A	109.5
C8—C9—C10	118.61 (19)	C30—C31—H31B	109.5
C8—C9—H9A	120.7	H31A—C31—H31B	109.5
C10—C9—H9A	120.7	C30—C31—H31C	109.5
C9—C10—C5	120.31 (19)	H31A—C31—H31C	109.5
C9—C10—N2	129.93 (19)	H31B—C31—H31C	109.5
C5—C10—N2	109.74 (18)	O2—C32—N5	120.66 (19)
C6—C11—H11A	109.5	O2—C32—C28	120.42 (19)
C6—C11—H11B	109.5	N5—C32—C28	118.92 (18)
H11A—C11—H11B	109.5	N5—C33—C34	112.4 (2)
C6—C11—H11C	109.5	N5—C33—H33A	109.1
H11A—C11—H11C	109.5	C34—C33—H33A	109.1
H11B—C11—H11C	109.5	N5—C33—H33B	109.1
O1—C12—N6	120.5 (2)	C34—C33—H33B	109.1
O1—C12—C8	120.5 (2)	H33A—C33—H33B	107.9
N6—C12—C8	119.02 (19)	C33—C34—C35	114.4 (2)
N6—C13—C14	113.7 (2)	C33—C34—H34A	108.7
N6—C13—H13A	108.8	C35—C34—H34A	108.7
C14—C13—H13A	108.8	C33—C34—H34B	108.7
N6—C13—H13B	108.8	C35—C34—H34B	108.7
C14—C13—H13B	108.8	H34A—C34—H34B	107.6
H13A—C13—H13B	107.7	C36—C35—C40	117.3 (3)
C13—C14—C15	110.8 (2)	C36—C35—C34	121.0 (3)
C13—C14—H14A	109.5	C40—C35—C34	121.7 (3)
C15—C14—H14A	109.5	C35—C36—C37	121.8 (4)
C13—C14—H14B	109.5	C35—C36—H36A	119.1
C15—C14—H14B	109.5	C37—C36—H36A	119.1
H14A—C14—H14B	108.1	C38—C37—C36	119.2 (4)
C16—C15—C20	117.6 (2)	C38—C37—H37A	120.4
C16—C15—C14	120.9 (2)	C36—C37—H37A	120.4
C20—C15—C14	121.5 (3)	C39—C38—C37	120.2 (4)
C15—C16—C17	121.7 (3)	C39—C38—H38A	119.9
C15—C16—H16A	119.1	C37—C38—H38A	119.9
C17—C16—H16A	119.1	C38—C39—C40	120.4 (4)
C18—C17—C16	120.1 (3)	C38—C39—H39A	119.8
C18—C17—H17A	119.9	C40—C39—H39A	119.8
C16—C17—H17A	119.9	C35—C40—C39	121.1 (4)
C19—C18—C17	118.7 (3)	C35—C40—H40A	119.5
C19—C18—H18A	120.6	C39—C40—H40A	119.5
C17—C18—H18A	120.6	C4—N1—C5	107.75 (17)
C18—C19—C20	121.4 (3)	C4—N1—H1	122.7 (16)
C18—C19—H19A	119.3	C5—N1—H1	126.8 (16)
C20—C19—H19A	119.3	C4—N2—C10	105.38 (17)
C19—C20—C15	120.4 (3)	C24—N3—C25	104.92 (16)
C19—C20—H20A	119.8	C24—N4—C26	107.21 (17)
C15—C20—H20A	119.8	C24—N4—H4	127.3 (15)
C22—C21—H21A	109.5	C26—N4—H4	125.2 (15)
C22—C21—H21B	109.5	C32—N5—C33	122.83 (19)
H21A—C21—H21B	109.5	C32—N5—H5	118.8 (17)
C22—C21—H21C	109.5	C33—N5—H5	117.7 (17)
H21A—C21—H21C	109.5	C12—N6—C13	120.33 (19)
H21B—C21—H21C	109.5	C12—N6—H6	121.9 (17)
C21—C22—C23	113.4 (4)	C13—N6—H6	116.7 (17)
			
C1—C2—C3—C4	−179.9 (2)	C27—C28—C29—C30	0.8 (3)
C2—C3—C4—N2	7.6 (3)	C32—C28—C29—C30	−178.92 (19)
C2—C3—C4—N1	−173.1 (2)	C28—C29—C30—C25	−0.7 (3)
N1—C5—C6—C7	178.2 (2)	C28—C29—C30—C31	178.8 (2)
C10—C5—C6—C7	−3.5 (3)	C26—C25—C30—C29	−0.1 (3)
N1—C5—C6—C11	−3.2 (4)	N3—C25—C30—C29	179.91 (19)
C10—C5—C6—C11	175.1 (2)	C26—C25—C30—C31	−179.6 (2)
C5—C6—C7—C8	1.3 (3)	N3—C25—C30—C31	0.4 (3)
C11—C6—C7—C8	−177.3 (2)	C27—C28—C32—O2	−1.8 (3)
C6—C7—C8—C9	1.1 (3)	C29—C28—C32—O2	177.9 (2)
C6—C7—C8—C12	−177.6 (2)	C27—C28—C32—N5	178.43 (19)
C7—C8—C9—C10	−1.4 (3)	C29—C28—C32—N5	−1.9 (3)
C12—C8—C9—C10	177.36 (18)	N5—C33—C34—C35	−173.9 (2)
C8—C9—C10—C5	−0.7 (3)	C33—C34—C35—C36	68.7 (4)
C8—C9—C10—N2	−178.6 (2)	C33—C34—C35—C40	−112.2 (3)
N1—C5—C10—C9	−177.99 (18)	C40—C35—C36—C37	−1.1 (5)
C6—C5—C10—C9	3.3 (3)	C34—C35—C36—C37	178.1 (3)
N1—C5—C10—N2	0.3 (2)	C35—C36—C37—C38	−0.4 (6)
C6—C5—C10—N2	−178.35 (19)	C36—C37—C38—C39	1.4 (7)
C9—C8—C12—O1	−1.1 (3)	C37—C38—C39—C40	−0.7 (7)
C7—C8—C12—O1	177.6 (2)	C36—C35—C40—C39	1.7 (5)
C9—C8—C12—N6	−178.74 (19)	C34—C35—C40—C39	−177.5 (3)
C7—C8—C12—N6	0.0 (3)	C38—C39—C40—C35	−0.9 (6)
N6—C13—C14—C15	177.0 (2)	N2—C4—N1—C5	−1.6 (2)
C13—C14—C15—C16	82.9 (3)	C3—C4—N1—C5	179.02 (19)
C13—C14—C15—C20	−93.9 (3)	C6—C5—N1—C4	179.2 (2)
C20—C15—C16—C17	0.8 (4)	C10—C5—N1—C4	0.7 (2)
C14—C15—C16—C17	−176.1 (3)	N1—C4—N2—C10	1.8 (2)
C15—C16—C17—C18	0.1 (5)	C3—C4—N2—C10	−178.9 (2)
C16—C17—C18—C19	−1.7 (5)	C9—C10—N2—C4	176.8 (2)
C17—C18—C19—C20	2.3 (6)	C5—C10—N2—C4	−1.3 (2)
C18—C19—C20—C15	−1.4 (6)	N4—C24—N3—C25	−0.2 (2)
C16—C15—C20—C19	−0.1 (5)	C23—C24—N3—C25	177.0 (2)
C14—C15—C20—C19	176.8 (3)	C26—C25—N3—C24	0.2 (2)
C21—C22—C23—C24	−67.5 (4)	C30—C25—N3—C24	−179.8 (2)
C22—C23—C24—N3	142.9 (3)	N3—C24—N4—C26	0.2 (2)
C22—C23—C24—N4	−40.1 (3)	C23—C24—N4—C26	−177.1 (2)
N3—C25—C26—N4	−0.1 (2)	C27—C26—N4—C24	179.0 (2)
C30—C25—C26—N4	179.95 (18)	C25—C26—N4—C24	−0.1 (2)
N3—C25—C26—C27	−179.21 (18)	O2—C32—N5—C33	4.5 (3)
C30—C25—C26—C27	0.8 (3)	C28—C32—N5—C33	−175.66 (19)
N4—C26—C27—C28	−179.6 (2)	C34—C33—N5—C32	95.4 (3)
C25—C26—C27—C28	−0.7 (3)	O1—C12—N6—C13	−2.0 (3)
C26—C27—C28—C29	−0.1 (3)	C8—C12—N6—C13	175.6 (2)
C26—C27—C28—C32	179.64 (17)	C14—C13—N6—C12	−156.5 (2)

### Hydrogen-bond geometry (Å, º)

**Table:**

*D*—H···*A*	*D*—H	H···*A*	*D*···*A*	*D*—H···*A*
N1—H1···O2^i^	0.88	1.91	2.744 (2)	157
N4—H4···N2^ii^	0.89	2.01	2.882 (3)	165
N5—H5···O1	0.88	2.06	2.849 (4)	148
N6—H6···N3^iii^	0.88	2.25	3.109 (2)	169

Symmetry codes: (i) *x*+1, *y*+1, *z*; (ii) −*x*, −*y*+1, −*z*; (iii) −*x*+1, −*y*+1, −*z*.
